# Behavioural therapy for smoking cessation: The effectiveness of different intervention types for disadvantaged and affluent smokers^☆^

**DOI:** 10.1016/j.addbeh.2013.07.010

**Published:** 2013-11

**Authors:** Rosemary Hiscock, Susan Murray, Leonie S. Brose, Andy McEwen, Jo Leonardi Bee, Fiona Dobbie, Linda Bauld

**Affiliations:** a Department for Health, University of Bath, BA2 7AY, UK; b School of Management and UK Centre for Tobacco Control Studies, University of Stirling, Stirling FK9 4LA, UK; c National Centre for Smoking Cessation and Training (NCSCT), University College London, 1-19 Torrington Place, London WC1E 7HB, UK; d Cancer Research UK, Health Behaviour Research Centre, University College London, Gower Street, London WC1E 6BT, UK; e Faculty of Medicine & Health Sciences, University of Nottingham, Nottingham City Hospital, Hucknall Road, Nottingham NG5 1PB, UK

**Keywords:** GP, General Practitioner (family doctor), HCA, Health Care Assistant: undertakes routine care tasks such as: temperature and pulse rate, maintaining standards of hygiene, helping with patient mobility and emotional support, under the supervision of qualified nurses (NHS Scotland, 2010), IMD, Index of Multiple Deprivation (England), NCSCT, National Centre for Smoking Cessation & Training, NHS, National Health Service (UK), NRT, Nicotine Replacement Therapy, NS-SEC, National Statistics Socio-Economic Classification (UK) (in this analysis ‘retired’ were merged with ‘caring for home’ and ‘full time students’ were merged with ‘unclassified’ to differentiate socio-economic status from age), PCT, Primary Care Trust (English health administration areas — there are ~ 150 PCTs serving a population of 56 million people), SES, Socio-economic status, SSS, Stop Smoking Services, Smoking cessation, Socio-economic status, Health disparities, Open groups, Closed groups, Specialist

## Abstract

**Background:**

Disadvantaged smokers are less likely to be successful when trying to stop smoking than more affluent smokers. In the UK, NHS Stop Smoking Services (SSS) provide a range of pharmacotherapy and behavioural support, delivered by advisors with a range of backgrounds. Whether the types of support provided and who provides it influence differences in quit rates amongst low SES smokers compared with high SES smokers has not previously been examined.

**Methods:**

202,084 records of smokers in England who attended a NHS Stop Smoking Service between July 2010 and June 2011 were acquired. Smokers were followed-up by services at four weeks post quit date. Multilevel logistic regression models of CO validated quits were employed. Disadvantage was explored through the National Statistics Socio-Economic Classification (NS-SEC) and by eligibility for free prescriptions, an indicator of low income amongst adults aged between 19 and 59 in England.

**Results:**

Affluent smokers were more likely to quit than disadvantaged smokers (OR 1.38 (1.35 to 1.42) for clients who paid for prescriptions compared to those eligible for free prescriptions). 80% of service clients received one-to-one counselling but open group forms of behavioural therapy were more successful (main effect OR 1.26 (1.12 to 1.41)) except amongst some of the most disadvantaged clients (long-term unemployed and prisoners). Closed groups were little deployed and they were not significantly more successful than one-to-one behavioural therapy after controls. Who delivered treatment did make a difference for some clients, with all but the most affluent less likely to be successful if they had been treated by a nurse compared with other types of advisers, including smoking cessation specialists (main effect OR 0.73 (0.65 to 0.83)).

**Conclusion:**

This study provides further evidence that disadvantaged smokers find quitting more difficult even when they have attended a smoking cessation programme. The findings suggest that open groups should be promoted, although they may not be as effective as other forms of behavioural therapy for the long-term unemployed or prisoners. Further research is required to explore why most groups of smokers who attended services staffed by nurses were less likely to quit than those who received treatment from other types of advisors.

## Highlights

• Higher socio-economic status was associated with quitting smoking
• Open groups were usually the most effective intervention type
• Open groups were not more effective for prisoners and the unemployed
• After controls, closed groups were not more effective than one to one support
• Generally nurse advisors were less successful than other advisor types

## 1 Introduction

The disease and mortality burden from smoking falls heaviest on low socio-economic status (SES) smokers (Kunst, Giskes, & Mackenbach, 2004; Mackenbach et al., 2008). While smoking rates have declined in the developed world, the decline has been slower or non-existent amongst low SES groups in these countries. This means that inequalities in smoking rates and inequalities in smoking-related conditions have increased (Barnett, 2000; Dube, Asman, Malarcher, & Caraballo, 2009; Federico, Kunst, Vannoni, Damiani, & Costa, 2004; Fernandez et al., 2001; Jarvis, 1994; Jefferis, Power, Graham, & Manor, 2004; La Vecchia, Decarli, & Pagano, 1986; Peretti-Watel, Seror, Constance, & Beck, 2009). The desire to quit smoking is equal across SES groups but success is not (Kotz, Fidler, & West, 2009; Reid, Hammond, Boudreau, Fong, & Siahpush, 2010). Disadvantaged smokers are less likely to quit than other smokers for a variety of reasons such as: reduced social support for quitting; lower motivation to quit; stronger addiction to tobacco; increased likelihood of not completing courses of pharmacotherapy or behavioural support sessions; psychological differences such as lack of self-efficacy and tobacco industry marketing (Hiscock, Bauld, Amos, Fidler, & Munafo, 2011). This pattern is true for those attempting to quit alone and those quitting with help from a smoking cessation programmes such as the UK's national network of services, known as NHS Stop Smoking Services (SSS) (Hiscock, Judge, & Bauld, 2011; Kotz et al., 2009; NICE, 2008).

NHS Stop Smoking Services were set up in 1999 to reduce smoking-related deaths particularly from cancer and coronary heart disease (NHS Executive, 1999) which predominantly occur amongst disadvantaged groups (Jha et al., 2006). SSS routinely collect self-reported outcomes at 4 weeks post quit date and biochemically validate this by conducting a breath test for levels of carbon monoxide (CO) (Murray et al., 2012). Previous studies have shown that the services are effective and cost effective (Bauld et al., 2012; Flack, Taylor, & Trueman, 2006; Godfrey, Parrott, Coleman, & Pound, 2005). The SSS provide a range of pharmacotherapy and behavioural support, delivered by advisors with a range of backgrounds. Whether the types of support provided and who provides treatment influences differences in quit rates between low SES and high SES smokers has not been examined and is explored in this paper.

### 1.1 Interventions delivered

NHS SSS provide support in the form of behavioural therapy either through one-to-one sessions, drop-ins or in groups typically involving weekly support over a period of at least 4 weeks after a quit date is set (NICE, 2008). Behavioural therapy and cognitive behavioural therapy are evidence-based treatments which aim to help people achieve specific aims or goals, such as smoking cessation, through focussing on the current situation rather than the past (Association for Behavioural and Cognitive Therapies, 2008; Guichenez et al., 2007). Compared to one-to-one treatment, group therapy offers the potential advantages of: receiving feedback from peers; modelling behaviour discussed by other group members; learning from the shared perspectives of group members; potentially increasing members' supportive social networks and reduced cost (Herkov, 2010; Paddock, Hunter, Watkins, & McCaffrey, 2011). However, whether better outcomes do occur in groups compared to one-to-one interventions requires more research (Cuijpers, van Straten, & Warmerdam, 2008; Tucker & Oei, 2007). There are two primary forms of group behavioural therapy: closed groups and open groups (Psychiatric Nursing, 2011).

Closed groups in the SSS involve structured, multi-session group courses with pre-arranged start and finish dates (Department of Health, 2007) starting with a minimum of eight clients (Department of Health, 2011) and are based on the scientifically validated ‘Maudsley model’ (Hajek, 1989; NHS Executive, 1999). Open groups deliver support in a flexible format where participants can choose when to attend; they have been used in a number of countries to provide support and treatment for a range of health problems or to facilitate behaviour change (Paddock et al., 2011; White, Bradley, Ferriter, & Hatzipetrou, 1998). Advantages of these open or rolling groups over closed groups, include: immediate starts (Morgan-Lopez, Saavedra, Hien, & Fals-Stewart, 2011; Ware & Bright, 2008); no fixed programme so clients can progress at their own pace and group leaders can be more responsive to individual client needs (Ware & Bright, 2008) and new clients learning from the experiences of those who have attended for longer (Ware & Bright, 2008). Thus, open groups have potential to be an effective form of behavioural therapy. There is however very little literature on the effectiveness of open groups compared with other forms of behavioural support (Bauld et al., 2012; Ware & Bright, 2008).

In 2010–2011, routine monitoring data from SSS in England suggested that from a total of 787, 527 clients, the majority (81%) received one-to-one structured support, 11% attended drop-in clinics, 3% attended drop-in rolling groups and 2% attended closed groups (NHS Information Centre, 2011). Telephone support, family or couple counselling and unclassified support were each received by 1% clients. However, outcomes at four weeks (post quit date) suggest that closed groups, despite being the least popular, are the most successful with an average quit rate of 60% for closed groups and 55% for rolling groups but only 49% for drop-ins and 48% for one-to-one support (NHS Information Centre, 2011). These patterns have also been found in longer term evaluations of the services (Bauld, Chesterman, Ferguson, & Judge, 2009; Bell et al., 2006; Brose et al., 2011) but there has been little attention paid to intervention type and disadvantage.

NHS SSS therapists (known as ‘advisors’) can be either specialists, who are employed only to work as smoking cessation advisors or can be people working in other health and social care roles such as: pharmacy employees; General Practitioners (GPs); nurses; Health Care Assistants (HCA); and midwives, who deliver stop smoking support as one part of their post. The type of therapist may influence uptake and effectiveness of treatment. Some potential clients are reluctant to access specialist services due to stigma or travel issues (Carter & Fairburn, 1995) and specialist staff may be more expensive to employ (Katon, Von Korff, Lin, & Simon, 2001). Furthermore, other health professionals, such as GPs, often encounter patients in routine practice with problems that could benefit from behavioural therapy (Leclerc, 1998). Evidence suggests that nurse conducted behavioural therapy can improve care and outcomes and it has been suggested that specialists should only handle complex cases (Katon et al., 2001).

Data on the type of SSS advisor is not collected routinely (NHS Information Centre, 2011) and there have not been any published recent evaluations (Bell et al., 2006). However there has been some work on the setting where the behavioural intervention takes place. A recent study of 24 English Services (Brose et al., 2011) found that the highest quit rates are in specialist clinics, these rates were significantly higher than primary care settings but not significantly higher than pharmacy or other settings. There is also evidence that inpatient interventions in hospitals are effective in facilitating quitting (Bell et al., 2006). In one study, a pharmacy intervention provided a low but still cost-effective quit rate (Bauld et al., 2012). Furthermore pharmacies have the potential to reach many smokers due to offering a range of locations and opening hours without the need for appointments.

SSS provide behavioural support in combination with one of three forms of pharmacotherapy — nicotine replacement therapy (NRT — sometimes used as combination therapy employing more than one product), bupropion (Zyban) or varenicline (Champix). In 2010–2011, the most widely used forms of pharmacotherapy provided by SSS were NRT (63%) and varenicline (31%) (NHS Information Centre, 2011). However, despite NRT being commonly used, the short-term (four week) quit rate for NRT (44%) was lower than varenicline (59%) and bupropion (52%) (Brose et al., 2011). Further evidence suggests that NRT is more effective when used in combination with behavioural support (NICE, 2008), but there is insufficient evidence to confirm whether NRT is more or less useful for disadvantaged or affluent smokers (Amos et al., 2011). However, previous research in the UK has shown that the use of smoking cessation programmes including behavioural support and pharmacotherapy can reduce inequalities if targeted towards disadvantaged groups (Chesterman, Judge, Bauld, & Ferguson, 2005).

The difference in success rates between low and high SES smokers begs the question of whether services could be optimised to help low SES smokers. Thus, the research questions explored in this paper are:

• Are some types of services less effective for low SES smokers than others?
• Do low SES smokers access more or less effective behavioural support?
• Is treatment for low SES smokers delivered by more or less effective advisor types?

## 2 Material and methods

English SSS store their routine monitoring data on databases often managed by external agencies. These databases can include more detailed information than the summary reporting required by the NHS. One of these databases, used in just under half of SSS in England, was used for this study. Forty nine of about sixty SSS who used the ‘Quit Manager’ database (North51, 2010) at the time of the study allowed their data to be used for research purposes and clients consent to their data to be used for research so ethical approval was not needed for this specific analysis. Six months of client records were downloaded in both January 2011 and July 2011 (n = 202,084). Records with quit dates in December 2010 (n = 7304) and June 2011 (n = 12,177) were excluded so that all clients had the potential to be followed up at 4 weeks. Records were also excluded if the client's age (n = 115), gender (n = 46) or advisor (n = 5573) were unknown, if they were not aged between 19 and 59 (n = 40,023) and if they were pregnant (n = 6606). This left 132,586 quit attempts in the analysis. Final numbers and numbers of excluded cases do not tally as a client record could be excluded for more than one reason.

### 2.1 Outcome

The main outcome variable was CO validated quit at 4 weeks post quit date (defined as self-report of not smoking in the past 2 weeks and having a CO reading of less than 10 ppm). Services are required to collect data on this short-term outcome by the UK government (Department of Health) and long-term quit rates can be estimated from this (Department of Health, 2011; Ferguson, Bauld, Chesterman, & Judge, 2005). In this dataset, the overall CO validation rate was 74% (Murray et al., 2012). An intention to treat approach was used, as is commonly the case in studies of smoking cessation, (West, Hajek, Stead, & Stapleton, 2005) so that clients lost to follow up were classified as continuing smokers. One stop smoking service had a markedly low CO validation rate (17% (Murray et al., 2012)) and was excluded from the analysis.

### 2.2 Disadvantage

The measures of individual disadvantage collected by the SSS are eligibility for free prescriptions and NS-SEC (National Statistics Socio-Economic Classification based on occupation and economic status) (ONS, 2008). NS-SEC was devised by the UK National Statistics agency and is used in the main UK government surveys (NICE, 2008). All citizens should be able to be classified by NS-SEC either by their occupation or by their reason for not having an occupation (such as being retired or permanently sick). Traditionally there has been an occupational division between those with non manual (white collar/bourgeoisie/middle class) occupations and manual occupations (blue collar/proletariat/working class respectively) (Marx & Engels, 1848) and this is reflected in SSS targets for quits amongst clients with routine and manual occupations (Bell et al., 2006). More recently there has also been concern about the development of an underclass of non working people (Myrdal, 1963) currently manifesting itself in the “welfare scroungers” debate (de Castella, 2012). NS-SEC can be used to differentiate these three groups.

Clients were also defined as low SES or disadvantaged if they were exempt from paying for NHS prescriptions for medicines and high SES if they paid for prescriptions. Free prescriptions are available for those on low income (NHS Business Services Authority, 2008a,b; NHS choices, 2011). Exemption can also be granted certain for medical reasons, pregnancy or having had a baby in the previous 12 months (NHS choices, 2011). There are additional age related exemptions for those who are 60 or over, below 16 year olds, and 16 to 18 year olds in full time education (NHS choices, 2011). Thus, the analysis was confined to non-pregnant clients aged between 19 and 59 at their quit date.

A level of disadvantage was allocated to each SSS through the Index of Multiple Deprivation (IMD) (www.communities.gov.uk, 2011) which gives every census area a score based on the level of disadvantage experienced by its population on a variety of different measures. These scores have been aggregated to health administration areas which have the same boundaries as Stop Smoking Services. Thus we were also able to measure whether disadvantage in the localities in which each SSS operate affect the rates of quitting.

In the analysis, NS-SEC categories were routine and manual, intermediate, managerial and professional, retired or home carer, sick or disabled and unable to work, long-term unemployed or never worked, in prison and other. Eligibility for free prescription categories were free prescriptions, pays for prescriptions and unknown. Disadvantage in the health service administration area served by the SSS was measured as the rank of average IMD 2010 score for service local health administration area (1–30, 31 to 60, 61 to 90, 91 to 120, 121 or higher (high is the least disadvantaged)).

### 2.3 Control variables

The following control variables were included: age at quit date quartiles (19 to 30, 31 to 39, 40 to 48 and 49 to 59), gender, ethnicity (Black, Asian, mixed or other; White; unknown), the month the quit date was set (tobacco control events, such as non smoking day occur at certain times of year and motivation varies with the seasons, for example, smokers may make quitting a new year's resolution and there may be a ‘back to school’ effect in September so the number of smokers setting quit dates with the SSS varies cyclically (Fox & Hampton, 2013); when quitters are more numerous, smokers may be more likely to be successful partly due to cohort effects/normalisation and because when there are more quitters, services are able to run more and larger groups), number of treatment episodes which refers to the number of quit dates set with the SSS (only episode, second episode, third episode, fourth or further episode) and medication (single NRT, combination NRT, bupropion only, varenicline only, mixed NRT/bupropion/varenicline, no medication or missing). These factors have previously been found to be associated with smoking cessation (Brose et al., 2011; Farren & Naidoo, 1996; King, Polednak, Bendel, Vilsaint, & Nahata, 2004; Quitline | Me Mutu, 2013).

### 2.4 Stop smoking service intervention variables

The following stop smoking service intervention descriptors were included: intervention type (one-to-one, drop-in clinic, open (rolling) group, closed group, other or missing) and SSS advisor (smoking cessation specialist, GP, nurse, health care assistant (HCA), pharmacy employee and other/missing).

## 3 Analysis

### 3.1 Are some types of services less effective for low SES smokers than others?

SPSS (version PASW 18.0) and Stata (StataCorp, 2010) were used for bivariate analysis. Co validated quit rates were calculated for all independent variables. Bivariate analysis however does not take into account other characteristics. Multilevel, multivariate logistic regression analysis was therefore conducted using MLwiN 2.24 (Rasbash, Charlton, Browne, Healy, & Cameron, 2009) with three levels: client record, advisor and SSS. At the time of the study SSS were provided by each health service administration area (termed Primary Care Trust (PCT)). A minority of neighbouring services shared advisors and these services were merged in the analysis so that the data had a completely nested structure.

Each fixed effect (independent variable that was not a level) was firstly added to the multilevel models separately (‘one fixed effect’ models), secondly all variables were added simultaneously firstly including NS-SEC to measure SES and secondly NS-SEC was removed and eligibility for free prescriptions was entered.

To determine whether some services were more appropriate for disadvantaged clients and other service types were more appropriate for more affluent clients, interactions were tested between low SES (as measured firstly by NS-SEC and secondly by eligibility for free prescriptions) and service type (measured by intervention type and advisor type).

Where an interaction term was found to be significant, cross-classified variables including most combinations of the categories of the two variables involved in the interaction were derived and included in the models (variables involved in the interaction were removed from the model). The reference group was rotated so that ‘one-to-one’ for each SES group became the reference category in turn. It was then possible to determine whether intervention type or advisor type differed significantly for each SES category. This technique has been used previously (Hiscock, Pearce, Blakely, & Witten, 2008).

### 3.2 Are low SES smokers accessing less effective behavioural support or services delivered by less effective advisor types?

Uptake rates and quit rates were tabulated for each intervention type and advisor type by free prescriptions and NS-SEC categories. Chi square tests were also undertaken.

## 4 Results

### 4.1 Are some types of services more effective for low SES smokers than others?

#### 4.1.1 Main effects: SES and service type

Service uptake, quit rates and odds ratios of quitting by SES and service type are described in Table 1. In ‘one fixed effect’ multilevel modelling affluent clients (those with managerial, professional and intermediate occupations) were significantly more likely to quit than clients with routine and manual occupations. However, in the multivariate model, prisoners were also more likely to quit. Clients classified as retired or home carers, permanently sick or disabled, long-term unemployed or never worked were less likely to quit. Respondents who paid for prescriptions were more likely to quit.

In the bivariate model and in the model where SES was measured by free prescriptions, clients who attended groups (both open and closed) were more likely to quit than those who received one-to-one behavioural support. When NS-SEC was included in the analysis there was no longer a significant difference between closed groups and one-to-one.

Clients who saw GPs and nurses were less likely to quit than clients who saw smoking cessation specialists in multilevel models. Clients who saw pharmacy employees were less likely to quit than clients who saw specialists in the bivariate model but not the multivariate models. Thus, GPs and nurses were less effective than specialists in the main effects models, although the number of clients treated by GPs was very small.

#### 4.1.2 Control variables

Uptake of services and chances of quitting for the control variables are described in Table 2. Each age quartile covered about 10 years. Similar numbers of men and women accessed the services (48.5% and 51.5% respectively). The ethnicity of those accessing the services was primarily white (88.2%). Clients came to the services from both disadvantaged and more affluent areas. The largest numbers of clients accessed the services between January and March. The majority of individuals were attending the services for the first time (64.6%), only 6.8% were on their fourth or more episode. Single NRT (22%), Combination NRT (36.3%) and Champix (28.7%) were commonly used to support quit attempts. Overall there were 132 586 cases in the analyses (described in Tables 1 and 2) and the overall CO validated quit rate was 34%.

The odds ratios of quitting for the control variables in the multilevel multivariate models changed little when NS-SEC was substituted for free prescriptions as the measure of SES. The odds of a successful quit attempt significantly increased as age increased. Gender had no significant effect on the quit rate. Whites were significantly more likely to have a successful quit attempt than other ethnic groups. Area deprivation did not predict quitting. Compared with July, those quitting in November had a lower chance of success and those quitting in January, February, September and October had a greater chance of success. Clients on their fourth or more quit attempt were more likely to succeed than those on their first attempt. Compared to single NRT, use of any other type of medication increased an individual's chance of a successful quit.

#### 4.1.3 Interactions

There were three significant interaction terms when the interaction between NS-SEC and intervention type was tested (p < .05): long-term unemployed/never worked and open groups, prisoners and open groups and prisoners and drop-ins. These were explored using a cross classified variables. Socio-economic status patterns were more or less retained (Table 5 Section 1a): for most intervention types, clients with professional and managerial occupations were significantly more likely to quit than clients with routine and manual occupations. Retired/home carers, long-term sick and long-term unemployed clients were less likely to quit for most interventions. Only for prisoners did the intervention type change the quit rate from significantly higher to significantly lower than the reference.

When the reference category was rotated (Table 5 Section 1b), open groups were more effective than one-to-one (significantly so for clients with routine and manual occupations and clients with professional and managerial occupations) except for the long-term unemployed and prisoners. For unemployed clients and prisoners, there was a significant difference in the direction of the relationship. Open groups were less effective than one-to-one, significantly so for prisoners. There were no significant associations for closed groups.

When the interaction between NS-SEC and advisor type was tested, there was one significant interaction term (p < .05): professional/managerial occupation and nurse advisor. Socio-economic status patterns were more or less retained (Table 5 Section 2a): only clients with higher grade occupations were sometimes significantly more likely to quit than the reference group (clients with routine and manual occupations who saw specialists). Retired/home carers, long-term sick and long-term unemployed clients who saw various advisor types were often significantly less likely to quit than the reference group.

For all disaggregated NS-SEC categories when the reference group was rotated (Table 5 Section 2b), clients who saw nurses were less likely to quit and this relationship was significant or approached significance for all categories except for clients with professional or managerial occupations. For clients with professional or managerial occupations, there was little difference according to advisor type.

Neither the interaction between eligibility for free prescriptions and intervention type nor the interaction between eligibility for free prescriptions and advisor type was significant.

### 4.2 Were disadvantaged clients receiving less effective services?

#### 4.2.1 Do low SES smokers access less effective behavioural support?

Smokers from all SES classifications accessed all intervention types. The largest number of quit attempts was made by clients with routine and manual occupations, over 36,000 (Table 4). More than 20,000 were made by clients with managerial and professional occupations with just 2000 made by the smallest NS-SEC category: prisoners. In terms of the types of behavioural support received, the vast majority of clients received one-to-one counselling (around 80% of quit attempts). The least common intervention type was closed groups for all NS-SEC categories (2% of quit attempts overall) except for prisoners. There were only about 2000 prisoners in the sample however.

More than 10,000 more quit attempts were made by clients who were eligible for free prescriptions than those who paid for prescriptions. Patterns were similar to NS-SEC.

Permanently sick clients were more likely to access the most successful intervention, open groups, than long-term unemployed clients (p < .001) and clients who paid for prescriptions were significantly more likely to access open groups that clients who were eligible for free prescriptions (p = .003).

#### 4.2.2 Are low SES smokers supported by less effective advisor types (bivariate analysis)?

Significantly more clients were seen by specialist advisors (56%) than GPs (3%) (Table 5), with the other advisor types falling in between these two categories. This pattern was true for all SES categories. There were similar uptake patterns when SES was measured by eligibility for free prescriptions.

#### 4.2.3 Were disadvantaged clients receiving less effective services?

The most effective intervention type across the sample as a whole was open groups (Table 1). It was not the case that open group support was markedly less frequent amongst disadvantaged NS-SEC groups (Table 3). The least effective advisor type was nurses (Table 1). It was not the case that disadvantaged groups were more likely to see nurses (Table 4).

## 5 Discussion

The UK's Stop Smoking Services have played an important role in efforts to reduce tobacco use in the UK for almost 15 years (Bauld et al., 2012; Flack et al., 2006; Godfrey et al., 2005). The average impact of the services included in this analysis was 152 extra ex-smokers per 100,000 population between mid-2010 and mid-2011 (Murray et al., 2012). In general, quit rates in this study followed those reported in previous research: more affluent smokers, older clients and clients taking varenicline or combination therapy were most likely to quit (Brose et al., 2011; Ferguson et al., 2005). Overall, the effectiveness of different interventions offered by the services did not differ significantly for clients from more or less affluent groups. For most clients, the most effective form of behavioural support was open groups. In terms of advisor type, clients who saw nurses were generally less likely to quit than clients who were treated by other advisor types.

Examining in more detail any differences between intervention types in the study, the form of behavioural support with the most success was open, rolling groups. The exception was for the long-term unemployed and the very small number (n = 2000) of prisoners in the study, for whom open groups were less effective than one-to-one counselling. There are a number of possible explanations for this difference, including the fact that for the long-term unemployed, for example, engaging with interventions that require interpersonal skills may be challenging or uncomfortable (McQuaid, Green, & Danson, 2005; Niepel, 2010).

The professional background of the advisor delivering support made little difference to outcomes, with the exception of nurses who were found to be less effective than smoking cessation specialists when treating most groups of clients. Interestingly, clients who saw health care assistants (HCAs) had higher success rates despite these assistants working in the same settings (primary care premises) as nurses (model not shown OR main effect 1.27 (1.10 to 1.46)). These HCAs have fewer formal qualifications but may have more time to spend with clients. They may also be able to build rapport more effectively because they are more likely to be recruited from the same local communities as smokers themselves (Bosley & Dale, 2008; Lizarondo, Kumar, Hyde, & Skidmore, 2010; NHS Scotland, 2010; Petrova, Vail, Bosley, & Dale, 2010).

In addition, although clients supported by GPs can achieve high quit rates this association disappeared once the SSS where they worked was taken into account — only a very small number of SSS recorded having GPs as smoking cessation advisors. It is possible that the boundary was blurred between brief advice and more intense interventions in the reporting of these SSS.

Some differences in intervention effectiveness for SES groups were found when SES was measured by NS-SEC rather than free prescriptions. The advantage of NS-SEC is that it provides a more finely graded distinction between SES groups which may be necessary to tease out whether and where changes should be made to services. There was no evidence that disadvantaged smokers were less likely to quit because they were attending less effective services i.e. it was not the case that disadvantaged smokers were not accessing open groups or were predominantly receiving behavioural support from nurses.

### 5.1 Limitations of the study

This study only provides short-term outcomes because routine data, where follow up is conducted at four weeks, was analysed. The advantage of this short period was that less than 30% of cases were lost to follow up. In smoking cessation studies lost to follow up tends to be an indicator of relapse (West, 2006) so these cases could be retained in the analysis as non quitters avoiding selection bias. A particular limitation of routine data is that quality varies resulting in a lower level of accuracy than would be the case in a research study. Data on level of tobacco dependence and social support was limited to a few cases and could not be used in the analysis. Data on the advisor type was also poorly collected (it is not a national requirement for reporting) so further research is needed to corroborate our results on the relationship between who provides support and outcomes.

The naturalistic design has the advantage of real world observation but it meant that there was no control group so we cannot be certain whether using the services significantly increased quitting. Background quit rates (the chances of a smoker quitting with no help from the services) have however been estimated to be between 2% and 3% for 2006 (West, 2006) which is considerably lower than the 34% quit rate for this study.

We measured disadvantage by two proxies: NS-SEC and eligibility for free prescriptions. Both of these have weaknesses. Only personal NS-SEC was available which may not reflect the disadvantage of household members who are not the chief income earner. Secondly, NS-SEC can be difficult to classify. NS-SEC was recorded by advisors rather than researchers with expertise in classifying socio-economic status and the questions asked to ascertain NS-SEC were simpler than those used by government surveys. This may have caused some idiosyncrasies in the results — occasionally quit rates by intervention type for clients with routine and manual occupations were more similar to clients with professional and managerial occupations than to clients with intermediate occupations.

Treatment was free for all clients through the UK government funded National Health Service. In other countries more affluent clients might be asked to pay for treatment. Eligibility for free prescriptions can be determined by medical reasons as well as SES. The collection of data on medical conditions was not collected consistently or in enough detail for us to determine whether the free prescriptions were due to health or income reasons. We excluded pregnant women but data on whether women had a child under the age of one was not collected so we could not exclude such women. Eligibility for free prescriptions is only a measure of disadvantage for 19 to 59 year olds in England. Results for all age groups are presented elsewhere (Murray et al., 2012). In general, results were similar.

SSS level disadvantage did not significantly predict quitting. This may be because the health administration areas were too large or SSS disadvantage does not necessarily correlate with the disadvantage of individual clients. Ethnic minorities varied by health administration area and to increase the robustness of the analysis all ethnic minorities were combined, as the numbers were very small. In future research it would be helpful to analyse individual ethnic groups separately.

The therapy provided by SSS is not standardised and is delivered by a variety of advisor types. The UK's national centre for smoking cessation and training (NCSCT) is aware of this variation and has recently published on the techniques that the evidence suggests are most effective (Michie, Churchill, & West, 2011; West, Walia, Hyder, Shahab, & Michie, 2010).

It is likely that group dynamics affect the chances of successful smoking cessation for smokers who took part in groups (Paddock et al., 2011). Group dynamics are extremely complex to model particularly in an open group setting where members change from session to session (Morgan-Lopez et al., 2011; Paddock et al., 2011) and no attempt to assess these was included here. Thus standard errors may have been underestimated (Paddock et al., 2011). Different group dynamics may exist in groups with all low SES members, all high SES members or mixed membership. In future research focussing on socio-economic status and group dynamics may provide more insight into how to increase quit rates amongst more disadvantaged smokers taking part in open and closed groups.

Another concern with intervention types is that there are similarities between one-to-one and drop-ins, drop-ins and open groups, and open and closed groups. Thus these interventions may in practice lie on a continuum rather than be distinct entities. The same issues apply with age and SSS disadvantage. These continuous variables were divided into quartiles and quintiles to aid interpretation and allow the possibility of non linear effects.

There was significant variation between advisors and between health administration areas in the full multivariate model. Thus there must be more characteristics that can explain the variation which we were not able to model, which may have implications for the quit rates of low SES and high SES groups, such as differences in health administration areas' priorities, leadership, enthusiasm and cultures (Amos et al., 2011) and motivation, gender or rapport with clients for advisors (Hiscock, Moon, Pearce, Barnett, & Daley, 2012).

### 5.2 Conclusions

This study provides new insight into the types of behavioural support and smoking cessation service characteristics that are associated with biochemically validated quit rates amongst smokers from different socio-economic groups. As other studies have found, smokers who were more disadvantaged were less likely to be successful in their quit attempt than more affluent groups, even after accessing cessation services. With the exception of the most disadvantaged clients (the long-term unemployed and prisoners), smokers who attended open group therapy for smoking cessation were more successful than those who received one-to-one support. Clients were equally likely to quit if they received support to stop from staff trained as smoking cessation specialists, health care assistants or pharmacists, but less likely to quit if they were treated by nurses. Further research is required to explore in more detail how the background and training of those involved in delivering smoking cessation services may interact with client characteristics to affect outcomes.

## Role of funding sources

Funding for this study was provided by National Institute of Health Research (NIHR) Health Technology Assessment Programme grant number 09/161/01. The NIHR Health Technology Assessment Programme had no role in the study design, collection, analysis or interpretation of the data, writing the manuscript, or the decision to submit the paper for publication. RH was funded by an Economic and Social Research Council UKCTCS (UK Centre for Tobacco Control Studies) fellowship (RES-590-28-0004) whilst working on this study.

## Contributors

RH undertook much of the analysis and wrote the majority of the first draft and revision of the paper. RH was also a co-applicant on the grant used to fund this study. SM assisted RH with the analysis and the results section of the paper and commented on subsequent drafts. LSB prepared the dataset and contributed to drafting of the paper. AMcE is the Director of the National Centre for Smoking Cessation and Training which holds the dataset used in the analysis and was a co-applicant on the grant. He also revised the paper. JL-B advised on the statistics used in the paper and contributed to the paper. FD is the project manager for the ELONS study which provided the grounds for this paper and contributed to the revision of the paper. LB is the Principal Investigator on the grant, had the original idea for the study and contributed to the paper.

## Conflict of interest

AMcE receives a personal income from Cancer Research UK via University College London. He has received travel funding, honoraria and consultancy payments from manufacturers of smoking cessation products (Pfizer, GSK and Novartis). He also receives payment for providing training to smoking cessation specialists, receives royalties from books on smoking cessation and has a share in a patent of a nicotine delivery device. There are no other conflicts of interest.

## Figures and Tables

**Table 1:** Quit rates and multilevel modelling odds ratios for SES and SSS service type variables main effects and higher level variance.

	N	%	% Quit	Bivariate	Multivariate SES measured by eligibility for free prescriptions	Multivariate SES measured by NS-SEC
*NS-SEC*						
Routine and manual	37,768	28.5	37.3	1		1
Intermediate	11,539	8.7	37.6	1.09 (1.04 to 1.14)		1.10 (1.05 to 1.15)
Managerial and professional	21,893	16.5	39.9	1.20 (1.15 to 1.24)		1.17 (1.12 to 1.21)
Retired or home care	10,203	7.7	33.9	0.89 (0.85 to 0.94)		0.90 (0.86 to 0.95)
Permanently sick	10,166	7.7	29.3	0.72 (0.69 to 0.76)		0.67 (0.64 to 0.71)
Long-term unemployed	21,132	15.9	28.1	0.67 (0.64 to 0.70)		0.71 (0.68 to 0.74)
In prison	2038	1.5	42.1	1.09 (0.94 to 1.26)		1.69 (1.45 to 1.97)
Other	17,847	13.5	28.8	0.75 (0.72 to 0.78)		0.83 (0.79 to 0.87)

*Eligibility for free prescriptions*						
Exempt	65,710	49.6	31.9	1	1	
Pays	53,370	40.3	39.2	1.42 (1.39 to 1.46)	1.38 (1.35 to 1.42)	
Unknown	13,506	10.2	27.1	0.93 (0.88 to 0.97)	0.98 (0.93 to 1.03)	

*Intervention type*						
One-to-one	104,556	78.9	33.9	1	1	1
Drop-in clinic	18,765	14.2	33.9	1.03 (0.96 to 1.09)	0.98 (0.92 to 1.05)	1.00 (0.94 to 1.07)
Open (rolling) group	3844	2.9	50.8	1.44 (1.29 to 1.61)	1.28 (1.14 to 1.43)	1.26 (1.12 to 1.41)
Closed group	2145	1.6	49.1	1.35 (1.20 to 1.52)	1.20 (1.06 to 1.36)	1.11 (0.98 to 1.26)
Other or missing	3276	2.5	23.4	0.64 (0.57 to 0.71)	0.59 (0.53 to 0.66)	0.59 (0.53 to 0.65)

*Advisor type*						
Specialist advisor	40,234	30.3	38.0	1	1	1
GP	2640	2.0	35.9	0.61 (0.48 to 0.77)	0.58 (0.46 to 0.73)	0.59 (0.46 to 0.74)
Nurse	13,095	9.9	29.1	0.72 (0.64 to 0.81)	0.73 (0.64 to 0.82)	0.73 (0.65 to 0.83)
HCA	5604	4.2	34.6	0.88 (0.75 to 1.02)	0.92 (0.79 to 1.07)	0.92 (0.79 to 1.07)
Pharmacy	13,731	10.4	32.2	0.86 (0.77 to 0.96)	0.96 (0.86 to 1.07)	0.96 (0.86 to 1.07)
Other or unknown	57,282	43.2	33.4	0.85 (0.78 to 0.93)	0.93 (0.84 to 1.02)	0.92 (0.84 to 1.01)

					Variance (95% CI)	Variance (95% CI)
*SSS variance*					0.19 (0.10 to 0.28)	019 (0.10 to 0.28)
Practitioner ID variance					0.46 (0.43 to 0.49)	0.45 (0.42 to 0.48)

**Table 2:** Quit rates and multilevel modelling odds ratios and 95% CI for control variables in main effects models.

	N	%	% Quit	One fixed effect	Multivariate (prescriptions)	Multivariate (NS-SEC)
Total (regression constant)	132,586	100.0	34.4		0.14 (0.11 to 0.19)	0.17 (0.13 to 0.23)
*Age quartile*						
19–30	33,492	25.3	27.2	1	1	1
31–39	32,546	24.5	34.8	1.48 (1.43 to 1.54)	1.44 (1.39 to 1.50)	1.42 (1.37 to 1.48)
40–48	35,857	27.0	37.1	1.65 (1.60 to 1.71)	1.60 (1.54 to 1.66)	1.60 (1.54 to 1.65)
49–59	30,691	23.1	38.5	1.78 (1.72 to 1.84)	1.75 (1.69 to 1.81)	1.79 (1.73 to 1.86)

*Gender*						
Female	68,232	51.5	34.1	1	1	1
Male	64,354	48.5	34.7	1.02 (0.99 to 1.04)	1.00 (0.97 to 1.02)	1.02 (0.99 to 1.04)

*Ethnicity*						
Black, Asian, mixed, other	10,511	7.9	30.7	1	1	1
White	116,998	88.2	34.9	1.16 (1.11 to 1.22)	1.05 (1.00 to 1.11)	1.07 (1.02 to 1.13)
Unknown	5077	3.8	29.5	0.97 (0.89 to 1.05)	0.97 (0.89 to 1.05)	0.99 (0.91 to 1.07)

*SSS area deprivation (IMD)*						
≤ 30	27,312	20.6	31.6	1	1	1
31–60	27,174	20.5	37.9	1.36 (0.92 to 2.01)	1.27 (0.83 to 1.96)	1.24 (0.81 to 1.92)
61–90	12,792	9.6	31.5	1.08 (0.70 to 1.68)	1.07 (0.66 to 1.73)	1.06 (0.66 to 1.72)
91–120	41,837	31.6	37.4	1.47 (1.04 to 2.09)	1.17 (0.80 to 1.72)	1.16 (0.79 to 1.71)
121 + (least deprived)	23,471	17.7	29.6	1.37 (0.92 to 2.02)	1.28 (0.83 to 1.96)	1.26 (0.82 to 1.94)

*Quit month*						
July 2010	11,443	8.6	31.4	1	1	1
Aug 2010	10,406	7.8	33.1	1.07 (1.01 to 1.14)	1.05 (0.99 to 1.12)	1.05 (0.99 to 1.12)
Sept 2010	11,176	8.4	34.6	1.17 (1.10 to 1.24)	1.15 (1.08 to 1.22)	1.15 (1.08 to 1.22)
Oct 2010	11,677	8.8	34.0	1.13 (1.07 to 1.20)	1.09 (1.03 to 1.16)	1.09 (1.03 to 1.16)
Nov 2010	10,046	7.6	30.9	0.96 (0.91 to 1.03)	0.91 (0.86 to 0.97)	0.91 (0.86 to 0.97)
Jan 2011	20,495	15.5	39.0	1.39 (1.32 to 1.46)	1.36 (1.29 to 1.44)	1.36 (1.29 to 1.44)
Feb 2011	16,844	12.7	36.8	1.26 (1.19 to 1.33)	1.19 (1.13 to 1.26)	1.19 (1.13 to 1.26)
March 2011	16,744	12.6	33.6	1.09 (1.03 to 1.15)	1.03 (0.98 to 1.09)	1.04 (0.98 to 1.10)
April 2011	12,840	9.7	33.4	1.09 (1.03 to 1.16)	1.02 (0.96 to 1.09)	1.03 (0.97 to 1.09)
May 2011	10,915	8.2	31.9	1.02 (0.96 to 1.08)	1.00 (0.94 to 1.06)	1.00 (0.94 to 1.06)

*Episode*						
Episode 1	85,705	64.6	34.6	1	1	1
Episode 2	27,106	20.4	33.7	1.00 (0.97 to 1.03)	0.98 (0.95 to 1.01)	0.98 (0.95 to 1.01)
Episode 3	10,706	8.1	33.8	1.01 (0.97 to 1.06)	0.99 (0.94 to 1.04)	0.98 (0.94 to 1.03)
Episode 4 or more	9069	6.8	35.2	1.09 (1.04 to 1.15)	1.06 (1.01 to 1.11)	1.06 (1.01 to 1.12)

*Medication*						
Single NRT	29,126	22.0	25.1	1	1	1
Combination NRT	48,127	36.3	37.4	2.11 (2.03 to 2.19)	2.09 (2.02 to 2.17)	2.11 (2.04 to 2.19)
Zyban only	910	.7	31.9	1.57 (1.35 to 1.83)	1.45 (1.25 to 1.69)	1.45 (1.25 to 1.69)
Champix only	37,995	28.7	42.3	2.48 (2.38 to 2.57)	2.37 (2.28 to 2.47)	2.38 (2.29 to 2.47)
Mixed NRT/Zyban/Champix	3478	2.6	33.1	1.77 (1.63 to 1.92)	1.71 (1.58 to 1.86)	1.71 (1.58 to 1.86)
Missing or no medication	12,950	9.8	21.2	0.78 (0.74 to 0.83)	0.79 (0.74 to 0.84)	0.78 (0.74 to 0.84)

**Table 3:**
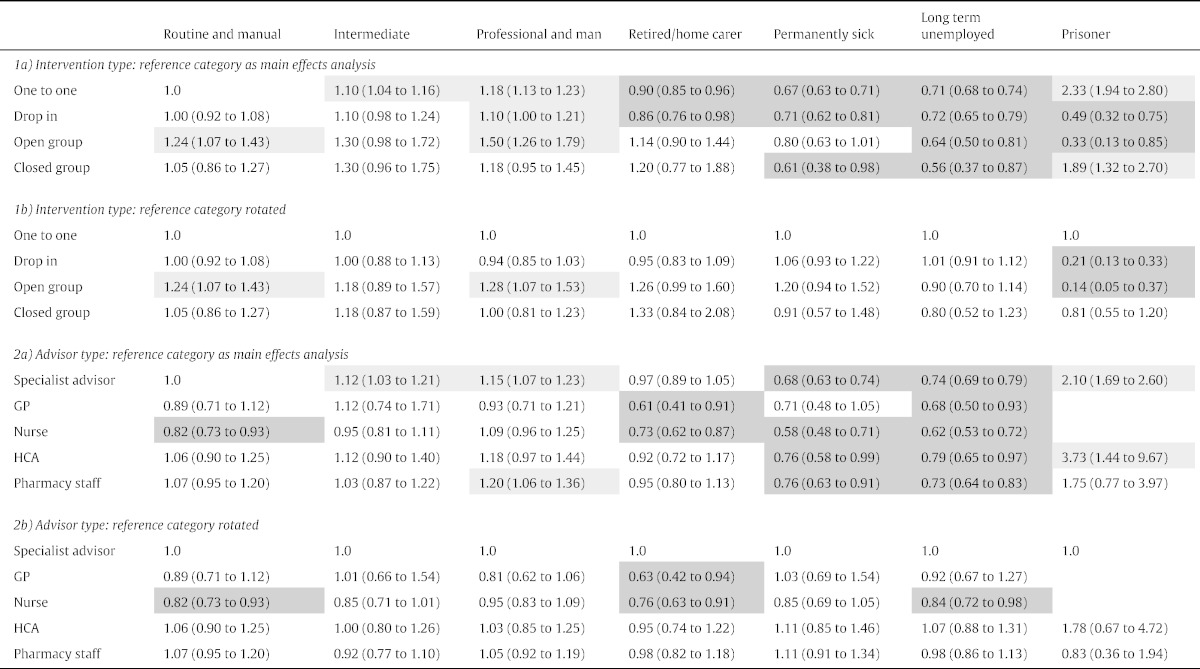
Multivariable, multilevel odds ratios (95% CI) for cross classified variables.

Also entered are SSS, advisor, age, gender, ethnicity, disadvantage in area covered by SSS, month quit-date set, number of treatment episodes, medication and advisor type (1a only), and intervention type (1b only). Intervention type: reference category as main effects analysis: other category 0.79 (0.75 to 0.83). Advisor type: reference category as main effects analysis: other category 0.87 (0.81 to 0.93). Bold font significant interaction, light shade: significantly more likely to quit, dark shade significantly less likely to quit (p < .05).

**Table 4:** Uptake and quit rates by intervention type and SES.

	Uptake		Uptake by intervention type^a^					CO validated quit at 4 weeks					
	N	%	One-to-one	Drop-in clinic	Open/rolling group	Closed group	P	One-to-one	Drop-in clinic	Open/rolling group	Closed group	Total	p
*NS-SEC*							< .001					P < .001	
Routine and manual	36,793	*33*	80%	15%	4%	2%	< .001	37%	37%	55%	51%	*37*%	< .001
Intermediate	11,211	*10*	80%	16%	2%	2%	< .001	38%	36%	49%	47%	*38*%	< .001
Managerial & professional	21,455	*19*	80%	15%	3%	2%	< .001	40%	38%	58%	51%	*40*%	< .001
Retired or home care	9944	*9*	80%	16%	4%	1%	< .001	34%	33%	53%	48%	*34*%	< .001
Sick or disabled and unable to work	9881	*9*	80%	15%	4%	1%	< .001	29%	30%	45%	38%	*29*%	< .001
Never worked or long-term unemployed	20,701	*19*	83%	15%	2%	1%	< .001	28%	27%	35%	33%	*28*%	< .010
In prison	2014	*2*	71%	12%	3%	14%	< .001	40%	40%	42%	57%	*42*%	< .001
*Total*	*111,999*	*100*	*80*%	*15*%	*3*%	*2*%	< .001	*35*%	*34*%	*51*%	*50*%	*36*%	<.*001*

*Prescriptions*							< .001					P <. 001	
Exempt	64,120	*55*	80%	16%	3%	1%	< .001	32%	31%	47%	44%	32%	< .001
Pays	52,073	*45*	81%	14%	3%	2%	< .001	39%	38%	55%	54%	40%	< .001
*Total*	*116,193*	*100*	*80*%	*15*%	*3*%	*2*%	< .001	35%	34%	51%	50%	35%	<.*001*

a The ‘other/unknown’ category was not included in chi square analysis.

**Table 5:** Uptake and quit rates by advisor type and SES.

	Uptake		Uptake by advisor type^a^						CO validated quit at 4 weeks						
	N	%	Specialist advisor	GP	Nurse	HCA	Pharmacy	p	Specialist advisor	GP	Nurse	HCA	Pharmacy	*Total*	p
*NS-SEC*								P < .001						P < .001	
Routine and manual	21,716	*33*	58%	4%	17%	7%	14%	< .001	41%	41%	31%	38%	35%	*38*%	< .001
Intermediate	6717	*10*	54%	2%	19%	9%	16%	< .001	39%	41%	35%	38%	35%	*37*%	0.017
Managerial and professional	12,632	*19*	52%	4%	18%	7%	20%	< .001	43%	43%	37%	41%	38%	*40*%	< .001
Retired or home care	6097	*9*	56%	3%	18%	8%	15%	< .001	38%	34%	28%	33%	32%	*35*%	< .001
Permanently sick	5874	*9*	64%	3%	14%	6%	14%	< .001	32%	35%	24%	31%	30%	*31*%	0.001
Long-term unemployed	11,406	*17*	52%	3%	16%	8%	21%	< .001	31%	30%	25%	29%	26%	*29*%	< .001
In prison	987	*2*	72%	0%	1%	7%	20%	< .001	48%	0%	5%	45%	24%	*43*%	NA
*Total*	65,429	*100*	56%	3%	17%	7%	17%	< .001	38%	39%	31%	36%	33%	*36*%	<.*001*

*Prescriptions*								< .001						P <. 001	
Exempt	37,313	*55*	56%	3%	13%	7%	21%	< .001	35%	33%	27%	31%	30%	*32*%	< .001
Pays	30,902	*45*	53%	4%	19%	8%	16%	< .001	43%	41%	34%	38%	36%	*40*%	< .001
*Total*	*68,215*	*100*	*55*%	*3*%	*16*%	*7*%	*19*%	< .001	*39*%	*38*%	*31*%	*34*%	*33*%	*36*%	<.*001*

a The ‘other/unknown’ category was not included in chi square analysis.
